# Graphene-Based Steganographic Aptasensor for Information Computing and Monitoring Toxins of Biofilm in Food

**DOI:** 10.3389/fmicb.2019.03139

**Published:** 2020-02-04

**Authors:** Qi Wang, Qingli Yang, Wei Wu

**Affiliations:** College of Food Science and Engineering, Qingdao Agricultural University, Qingdao, China

**Keywords:** aptasensor, graphene oxide, mycotoxin, fluorescence, encryption, steganography

## Abstract

Fungi-forming biofilm would produce various toxins in food. The toxin contamination will cause great harm to food and human health. Herein, a novel graphene-based steganographic aptasensor was assembled for multifunctional applications, which depended on the specific recognition and information encoding ability of DNA aptamers [mycotoxins, including zearalenone (ZEN) and ochratoxin A (OTA) aptamers, as models] and the selective absorption and fluorescence quenching capacities of graphene oxide (GO). The graphene-based steganographic aptasensor can be regarded as an information encryption and steganographic system using GO as a cover, aptamers for specific target recognition as information carriers and dual targets (ZEN and OTA) as special keys. In our work, the fluorescence of capture probes (Cy3 aptamer and Alexa Fluor 488 aptamer) was quenched by GO to realize information encryption. In the presence of dual targets in the GO–APT solution, Cy3 aptamer (APT1), and Alexa Fluor 488 aptamer (APT2) were released from the surface of GO, decrypting the hidden information. In addition, our work offers a sensor for rapid and sensitive simultaneous fluorescence determination of ZEN and OTA. The detection limit of the aptasensor was 1.797 ng/ml for ZEN and 1.484 ng/ml for OTA. In addition, the graphene-based steganographic aptasensor can be used to construct a molecular logic gate system in which GO, aptamers, and mycotoxins are employed as the input and compounds and fluorescence signals were used as the output. This would be helpful to control the biofilm toxin in the future.

## Introduction

Biofilm is an extracellular matrix (including toxin) secreted by biological flora, which is easy to adhere to a biological or non-biological surface (Galié et al., 2018; Xu et al., 2019). In other words, biofilm is a self-protection mechanism of bacteria and fungi. Once biofilm is formed, it is difficult to remove. Fungi can avoid being damaged by high temperature and pressure in the closed environment formed by a biofilm. In these circumstances, fungi will produce more mycotoxins to be discharged outside the biofilm. Mycotoxins are a kind of low-molecular-weight natural secondary metabolites that possesses strong toxicities to humans and animals (Rong et al., 2019). Therefore, the existence of biofilms will aggravate food pollution and other safety problems. Mycotoxins should be detected before biofilm formation to reduce food pollution.

Mycotoxin toxicity is mainly manifested as the following: damage to liver and kidney, teratogenicity, cancer, and mutation (Hussein and Brasel, 2001). Worst of all, mycotoxins not only lessen crop yield and quality but also cause significant economic losses (Turner et al., 2009; Sun X. D. et al., 2017). So far, the traditional detection methods of ochratoxin A (OTA) and zearalenone (ZEN) mainly include thin-layer chromatography (TLC), high-performance liquid chromatography (HPLC), gas chromatography (GC), capillary electrophoresis (CE), and enzyme-linked immunosorbent assay (ELISA) (Li et al., 2013; Zhang et al., 2018b; Wu et al., 2019a). These detection approaches face challenges due to their time-consuming, high-cost, and low-sensitivity methods. Moreover, there is a great possibility that foods and animal feeds will be contaminated simultaneously with several mycotoxins. However, a single detection pattern will not provide comprehensive monitoring for food safety. Thus, it is necessary to strengthen the simultaneous detection technology for mycotoxins in food and feed. Moreover, it is essential to develop a novel, rapid, low-cost, sensitive, simultaneous detection biosensor for mycotoxins.

In this internet era of information (text, audio, video, and image data), information storage and cryptography have crucial roles for countries and enterprises. To ensure information security and prevent information leakage, the universally used methods are information encryption and information concealment (Zhu et al., 2019). However, the safety level of these methods is low. Consequently, it is essential to improve information security and exploit a novel encryption and steganographic approach for information security. More interestingly, the processes of molecular interactions and chemical reactions can allow operation and transmission of information. Inspired by nature, DNA-based information encryption and concealment have emerged. In addition, due to DNA-specific molecular recognition, information encoding and ultrahigh-information-density capabilities, DNA-based biosensors, information storage, computing, encryption, and steganography have received increasing attention (Ejike et al., 2017; Zhang et al., 2017; Zhou et al., 2018; Lopez et al., 2019). Moreover, the development of conventional logic circuits based on semiconductors has reached a bottleneck development stage because the heat capacity, volume, and operational speed of semiconductor materials are difficult to further improve. However, the above issues can be solved perfectly by DNA molecules. Therefore, traditional logic circuits will give way to molecular logic gates, and DNA is an optimum alternative to silicon (Liu et al., 2012; Zhu et al., 2019).

Aptamers are short oligonucleotide sequences, and the ones used in this study were selected from a nucleic acid random library using systematic evolution of ligands by exponential enrichments (SELEX) *in vitro* (Jayasena, 1999; Wu et al., 2019b). The three-dimensional structure of an aptamer depends on the base sequence, the length of the nucleotide sequence, and the environmental conditions (Hermann and Patel, 2000). Due to their three-dimensional structure, aptamers possess high specificity and affinity for targets. Therefore, aptamers are usually assembled with nanomaterials as biomolecular recognition components in aptasensors (Pehlivan et al., 2019). Optical aptamer sensors are the most common aptamer sensors because the optical signal of the reaction process can be easily detected. A GO fluorescence resonance energy transfer (FRET) platform is constructed by fluorescent-modified aptamers and GO, which can be an ideal candidate for mycotoxin detection (Yugender Goud et al., 2017). In recent years, GO has attracted much interest due to its superior fluorescence quenching property relative to other quenchers (Wu et al., 2012; Kim et al., 2017). According to the FRET principle, a fluorophore was used as a fluorescence donor, GO was used as a fluorescence acceptor, and the fluorescence was blocked by GO. GO-based fluorescence biosensors have been extensively applied in a variety of detection fields. In addition, the different measured targets include metal ions (Qian et al., 2015; Wu et al., 2019c), cells (Wang et al., 2010), proteins (Zhou et al., 2015), pathogens (Zhu et al., 2019), mycotoxins (Sun A. L. et al., 2017; Yugender Goud et al., 2017), and DNA and other small molecules (Wang et al., 2018; Zhao et al., 2019). It is interesting to note that GO has different adsorption capacities for single-stranded DNA (ssDNA), double-stranded DNA (dsDNA), and G-quadruplex (Sun A. L. et al., 2017). GO has an extraordinary adsorption capacity for single-stranded oligonucleotides because of π-π stacking. According to this property, a fluorescence switch-on detection system has a theoretical basis.

In our work, a graphene-based steganographic aptasensor was designed for multifunctional applications, simultaneous fluorescence detection of mycotoxins (ZEN and OTA), and information computing, encryption, and concealing. The graphene-based steganographic aptasensor mentioned here can also be utilized to construct a simple DNA molecule logic gate system in which materials are the input and the compounds and fluorescence produced by the material interactions act as dual outputs. Moreover, graphene-based steganographic aptasensors will offer a novel model for molecular information encrypting and concealing technology.

## Materials and Methods

### Reagents and Materials

OTA and ZEN were purchased from Pribolab Co., Ltd. (Qingdao, China, http://www.pribolab.com). GO was purchased from Xianfeng Nanomaterials Tech Co., Ltd. (Nanjing, China). The oligonucleotide sequences of aptamer 1 (APT1, for ZEN) and aptamer 2 (APT2, for OTA) were taken from previously reported literature (McKeague et al., 2014; Zhang et al., 2018b). Two aptamers were purchased from Sangon Biotechnology Co., Ltd. (Shanghai, China). DNA probes were modified with the fluorescence dyes (Cy3 and Alexa Fluor 488) on their 5^′^-end. The oligonucleotide sequences of APT1 and APT2 are shown in Table 1. The methanol used in the experiment was analytically pure reagent. Ultrapure water (18.25 MΩ/cm) was used in all experiments.

**Table 1:** Oligonucleotides used in this study.

	**Modification**	**Sequences (5′-3′)**
APT1	5′ Cy3 (for ZEN)	CTACCAGCTTTGAGGCTCGATCCAGCTTATTCAATTATACCAGCTTATTCAATTATACCAGC
APT2	5′ Alexa Fluor 488 (for OTA)	AGCCTCGTCTGTTCTCCCGGCGCATGATCATTCGGTGGGTAAGGTGGTGGTAACGTTGGGGAAGACAAGCAGACGT

All fluorescence spectra were scanned using a Hitachi F-2700 fluorescence spectrophotometer (Hitachi Ltd., Japan). The slit width was 5 nm, and the voltage of the photomultiplier tube was 700 V. Fluorescence emission spectra were collected at an excitation wavelength of 512 nm for APT1 and 499 nm for APT2. The height trace images of the atomic force microscope (AFM) were scanned using an SPM-9700 AFM (Shimadzu, Japan). The dried samples on mica sheets were scanned in phase imaging mode.

All experiments were repeated at least three times.

### Operation Process of Detecting Dual Targets of Biofilm

Freeze-dried powder of the aptamers was diluted to working concentration (1 μM) in phosphate buffer saline (PBS) (containing NaCl 136.89 mM, KCl 2.67 mM, Na_2_HPO_4_ 8.1 mM, KH_2_PO_4_ 1.76 mM, pH = 7.4). Then, 30-μl GO nanosheets (250 μg/ml) were homogeneously mixed separately with 20 μl of APT1, APT2, and APT 1&2 at room temperature for 5 min. Subsequently, different concentrations of ZEN and OTA (0, 1, 5, 10, 50, 100, and 500 ng/ml) were dropped in the GO–aptamer mixed solution, and the ultimate volume of the solution of 1 ml was achieved by adding PBS. The final liquid was incubated at 45°C for 1 h (reaction temperature optimization shown in **Figure 4**). The most important thing was that the whole experiment was conducted under dark conditions. Fluorescence intensity was measured using an F2700 fluorescence spectrophotometer. Samples (GO, GO–APT1, GO–APT2, GO–APT1–ZEN, and GO–APT2–OTA) after incubation in a water bath were centrifuged for 10 min at 8,000 × *g* to obtain GO nanosheets. Then, GO nanosheets were diluted to an appropriate concentration (5 μg/ml) with ultrapure water and dispersed by ultrasonication for 10 min. The height trace image was scanned using an SPM-9700 AFM. Each sample was parallel scanned 20 times.

### Detection of ZEN and OTA in Real Samples

To confirm the practicality of the graphene-based steganographic aptasensor, wine samples were used for testing in our study. The wine sample was purchased from a local market (RT-Mart). First, the sediment was removed by centrifugation for 10 min. In addition, the supernatant was adjusted to pH 7.4 and diluted 20-fold with PBS buffer. Then, ZEN and OTA at known concentrations were spiked in pretreated samples. According to the above operation process, wine samples were quantitatively detected using a fluorescence aptasensor.

## Results and Discussion

### The Principle of a Fluorescence Aptasensor to Simultaneously Detect OTA and ZEN of Biofilm

**Scheme 1:**
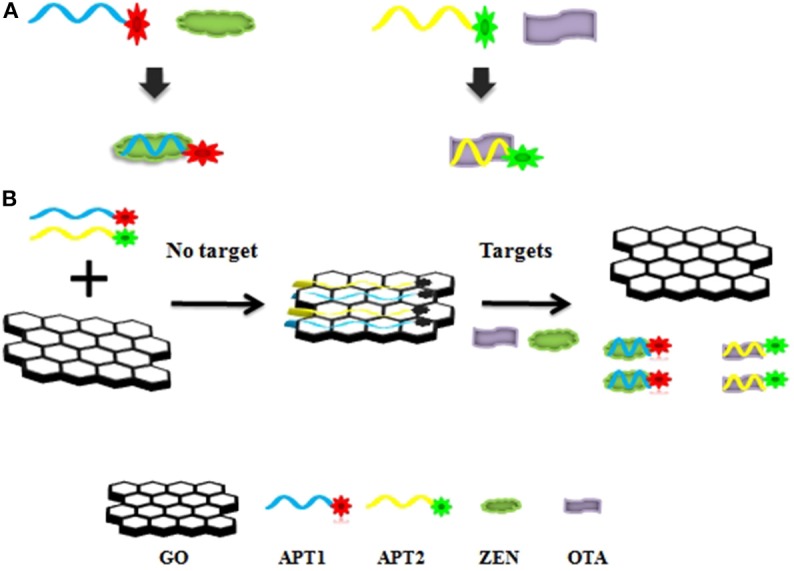
**(A)** Aptamer 1 (APT1) and aptamer 2 (APT2) and their specific targets zearalenone (ZEN) and ochratoxin A (OTA). **(B)** Schematic illustration of ZEN and OTA assay based on a fluorescence aptasensor using graphene oxide (GO) as a quencher.

The graphene-based steganographic aptasensor was composed of a ssDNA aptamer and GO. It depends on the high affinity and specific molecular recognition and information carrier abilities of the ssDNA aptamer and the fluorescence quenching and ssDNA adsorption capability of GO. The strategy of simultaneous detection of mycotoxins based on switch-on fluorescence aptasensors is shown in Scheme 1. Dual aptamers were regarded as prototypes, where APT1 can specifically recognize T1 (ZEN) and APT2 can specifically recognize T2 (OTA) (Scheme 1A, sequences in Table 1). In the simultaneous detection aptasensor system, APT1 and APT2 were adsorbed by GO due to the presence of oxygen-containing functional groups and conjugated structures on the GO surface. In addition, hydrogen bonds and π-π bonds formed between GO and APT1 and APT2. Accompanying the appearance of non-covalent bonds, the distance between GO and the aptamers decreased. In addition, the fluorescence of the aptamers was quenched by FRET. The GO-FRET platform was assembled by aptamers and GO. In the absence of the dual targets from the previous solution, the simultaneous detection system for mycotoxin was in the switch-off state. Upon the addition of targets, APT1, APT2, and their specific targets combine due to hydrogen bonding and electrostatic and hydrophobic interactions. The interaction forces of the aptamer target were more powerful than those of the GO aptamer (Zhang et al., 2018a). Subsequently, the aptamer target was released from GO, recovering the prominent fluorescence of APT1 and APT2. At this time, the simultaneous detection system of mycotoxin was in a switch-on state. Accordingly, with the increased concentration of targets, the fluorescence intensity recovery value increased. The target concentration and the restored fluorescence intensity show a good proportional relationship.

### Construction of a Logic Gate

**Figure 1:**
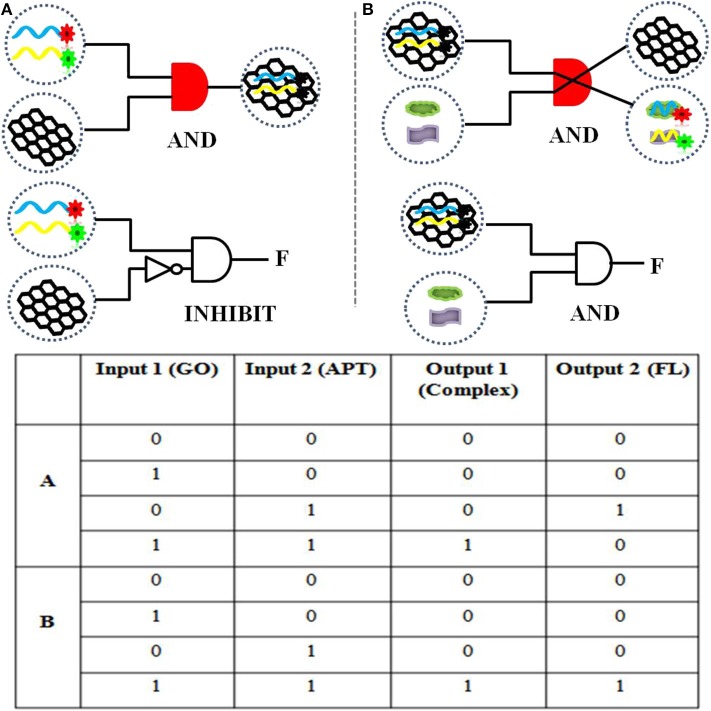
**(A)** The logical symbol diagram and truth table of the (aptamer “AND” GO) material gate and the “INHIBIT” (aptamer “AND” “NOT” GO) fluorescence gate. **(B)** The logical symbol diagram and truth table of the (aptamer–GO “AND” targets) matter gate and the (aptamer–GO “AND” targets) fluorescence gate.

To realize information computing, the feasibility of constructing a DNA logic gate was verified first. Our constructed APT–GO FRET platform can implement “AND” and “INHIBIT” logic gates by using changes in materials and energy. For the truth table of logic gates, 0 and 1 indicate the absence and presence of input and output, respectively. On the basis of the principle of the “AND” logic gate, the simultaneous existence of all the inputs induces the appearance of the output. This means that when the input is (1, 1), the output is 1. The output of an “INHIBIT” gate happens for one and only one specific input. In Figure 1, the materials (GO, APT, target, and compound) were regarded as the inputs and outputs of the integration circuit. The interaction of GO and APT (APT1/APT2/APT1&2) can be used to construct an “AND” logic gate. The GO–APT complex was present only when GO and APT simultaneously appeared in solution. When both inputs were 1, we marked the output as 1. In other cases, the output was 0. Simultaneous appearances of the dual targets (ZEN and OTA) and the GO-APT complex interacted with each other. Then, the transfer of the aptamer was accomplished from the surface of GO to the target, outputting the GO and APT target. Clearly, there is an “AND” logic gate for material exchange. On the basis of all cases in the truth table, the output was 1 when both inputs were 1. The interaction of matter leads to a change in fluorescence in the system. Therefore, fluorescence was deemed as the output. For the fluorescence signal, the self-construction of GO and aptamer can be understood as an “INHIBIT” logic gate. According to all cases of the truth table, fluorescence was outputted only when there were aptamers and no GO. In other words, when the inputs were (0, 1), the output was 1. With the combination of the GO–APT complex and dual targets, an “AND” logic gate will be formed, and fluorescence will be the output when both the GO-APT complex and dual targets exist.

The change of matter and energy can be used to realize molecular logical operation based on binaries 1 and 0. There is a close association between the biosensor and the logic gate. It is essential to further explore molecular logic gates with more complex combinations. Only in this way can molecular computing and molecular information technology be quickly realized.

### Verification of Experimental Feasibility

**Figure 2:**
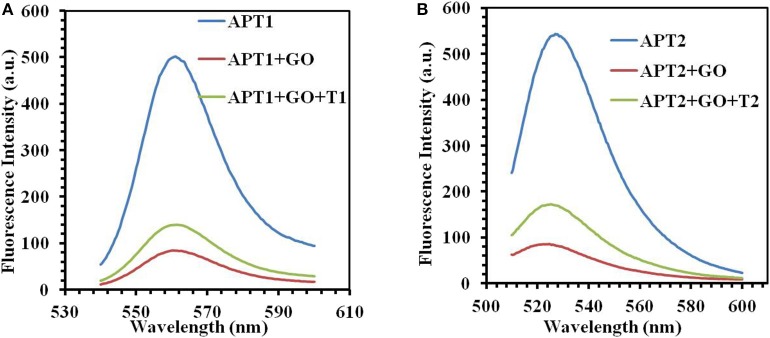
**(A)** The fluorescence emission spectra of APT1 for T1 (ZEN). **(B)** The fluorescence emission spectra of APT2 for T2 (OTA).

The next work tested and verified whether the principle of the switch-on fluorescence aptasensor was justified. The feasibility of the fluorescence aptasensor using GO as a quencher is demonstrated in Figure 2. The relationship among the fluorescence intensity of the aptamer, aptamer–GO, and aptamer–GO–target is shown clearly in Figure 2. The fluorescence intensity was stronger when there were aptamers alone (APT1 or APT2) in the solution (blue curves in Figures 2A,B). When the fluorescent aptamer was incubated with GO, the fluorescence intensity dropped sharply (blue and red curves in Figures 2A,B). The results showed that the fluorescence of the aptamers was blocked by GO. When the targets appeared in the aqueous solution, the aptamers (APT1 or APT2) combined with the special targets and were released from the GO surface, restoring the prominent fluorescence of the aptamers (red and green curves in Figures 2A,B). This phenomenon illustrated that the targets showed a stronger affinity than GO for the aptamers, resulting in APT1 and APT2 being released from GO and combining with the target. Therefore, the restored fluorescence intensity of the aptamer–target complexes was measured by fluorescence spectroscopy.

**Figure 3:**
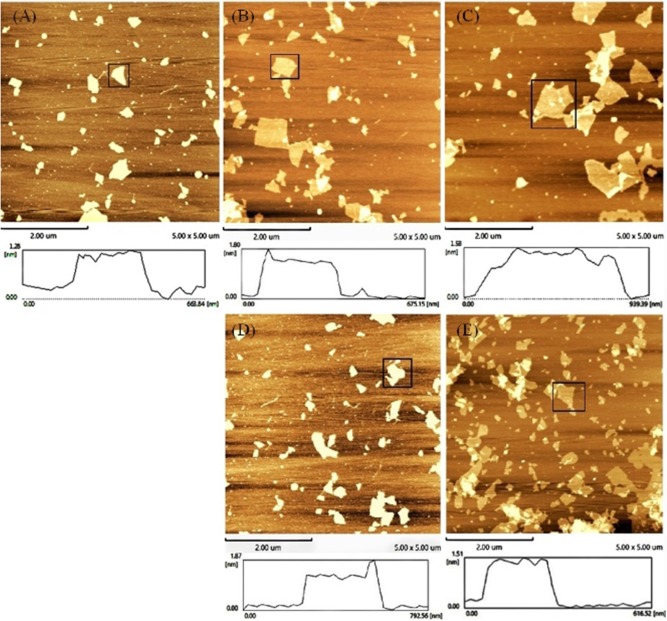
Atomic force microscope images of **(A)** GO, **(B)** GO–APT1, **(C)** GO–APT1–T1, **(D)** GO–APT2, and **(E)** GO–APT2–T2.

An AFM was also used to test the accuracy of the concept design. The height trace and size of GO were scanned by AFM. As shown in Figure 3, the average height of the GO sheets was approximately 1.28 ± 0.01 nm (Figure 3A). This result is consistent with the theory that GO is a single atomic layer. Then, the height of the aptamer–GO complexes was 1.80 ± 0.007 nm (Figure 3B) and 1.87 ± 0.034 nm (Figure 3D). The increased thickness demonstrated that aptamers were successfully adsorbed on the surface of GO. Finally, when the GO–APT complex was incubated with targets, its thickness significantly decreased to 1.58 ± 0.005 nm (Figure 3C) and 1.51 ± 0.008 nm (Figure 3E). This phenomenon confirmed that the aptamer was released from the surface of GO because the interaction forces of the aptamer target were stronger than those of the GO aptamer. Both of the above methods showed that the experimental design is correct.

### Temperature Optimization for Detection

To improve the efficiency of fluorescence recovery after adding targets, temperature optimization measures were taken. At room temperature, the fluorescence intensity did not recover significantly after adding the target. The interaction of GO and the aptamer was weakened by increasing the temperature, and the APT target desorbed. Thus, to enhance the fluorescence restoration, increasing the temperature is an ideal method. At the same time, excessively high temperatures will destroy the binding between the aptamer and the target. It is important to optimize the temperature.

**Figure 4:**
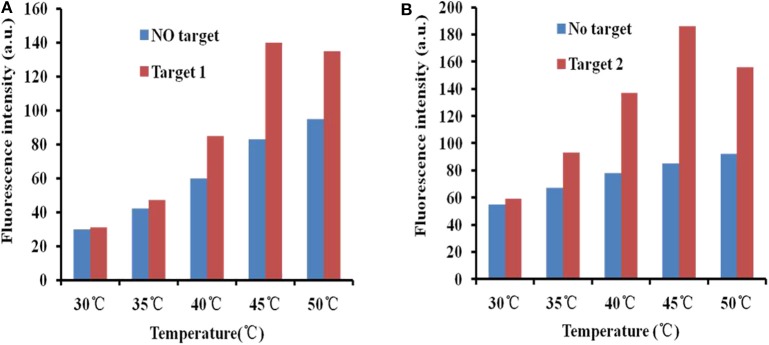
**(A)** Fluorescence desorption of the GO–APT complex in the presence and absence of T1 (500 ng/ml) at different temperatures (30–50°C). **(B)** Fluorescence desorption of the GO–APT complex in the presence and absence of T2 (500 ng/ml) at different temperatures (30–50°C).

The fluorescence intensity restoration increased with increasing temperature (30, 35, 40, 45, and 50°C) in general when there was no target (blue bars in Figure 4). This phenomenon confirmed the previous theory that high temperature accelerates the release of the aptamer from the GO surface. However, the phenomenon was different when targets were added to the GO–APT compound. The fluorescence signal increased regularly below 45°C (red bars in Figure 4). When the temperature exceeded 45°C, the fluorescence intensity restoration was weakened. Thus, 45°C is the critical point of the fluorescence intensity restoration.

### Sensitivity Test of the Fluorescence Aptasensor

**Figure 5:**
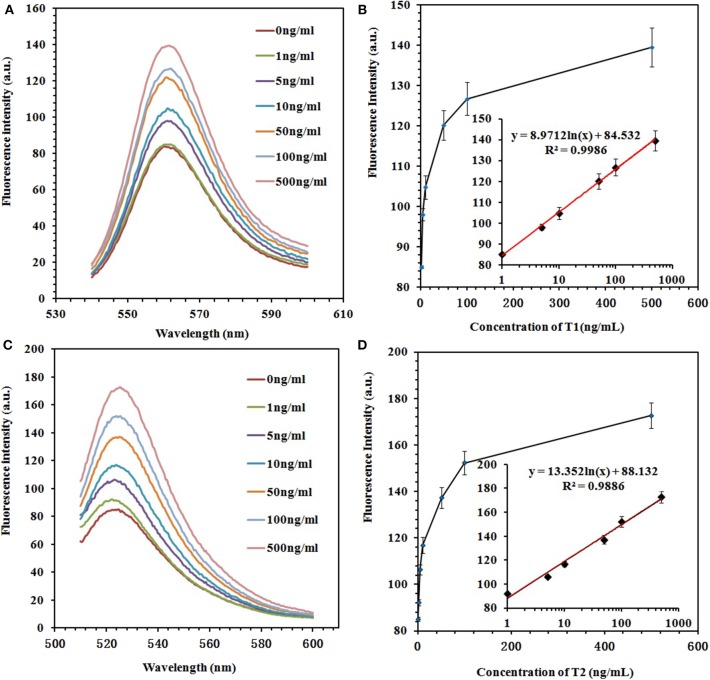
**(A)** Fluorescence emission spectra of the GO–APT1 complex (30 μg/ml; 20 nM) in the presence of ZEN (0, 1, 5, 10, 50, 100, and 500 ng/ml). **(B)** Calibration plot of ZEN with a concentration range of 1–500 ng/ml. **(C)** Fluorescence emission spectra of the GO–APT2 complex (30 μg/ml; 20 nM) in the presence of OTA (0, 1, 5, 10, 50, 100, and 500 ng/ml). **(D)** Calibration plot of OTA with a concentration range of 1–500 ng/ml.

To prove the sensitivity of the fluorescence-switch aptasensor, the targets (ZEN and OTA) at different concentrations (0, 1, 5, 10, 50, 100, and 500 ng/ml) were incubated with the GO–APT complex. The fluorescence intensity increased, as shown in Figure 5. With both increasing concentrations of ZEN and OTA, the fluorescence intensity increased from 83.97 to 139.6 (a.u.) for the GO–APT1 mixed solution and from 85.07 to 172.6 (a.u.) for the GO–APT2 mixed solution. As shown in Figure 5, the regression equation of ZEN was *y* = 8.9712 ln(*x*) + 84.532, *R*^2^ = 0.9986, and that of OTA was *y* = 13.352 ln(*x*) + 88.132, *R*^2^ = 0.9886. Hence, a conclusion was drawn that the target concentration and the restored value of fluorescence intensity have a good proportional relationship. The limit of detection (LOD) of this aptasensor is the target concentration responding to the fluorescence intensity of the mean blank value plus 3 standard deviations (SD). For ZEN, the mean fluorescent intensity of 20 blank experiments was 84.95, and the SD was 1.612. The responding fluorescence intensity of LOD was 89.786. According to standard equation (Figure 5B), LOD was 1.796 ng/ml. For OTA, the mean fluorescent intensity of 20 blank experiments was 87.37, and the SD was 2.01. The responding fluorescence intensity of LOD was 93.4. According to standard equation (Figure 5D), LOD was 1.484 ng/ml. The quenching and recovery of fluorescence illustrated that GO played a crucial role in the fluorescence switch aptasensor. A comparison of the many sensors for ZEN and OTA detection with the detection limits is summarized in Table S1.

### Specificity Test of the Fluorescence Aptasensor

**Figure 6:**
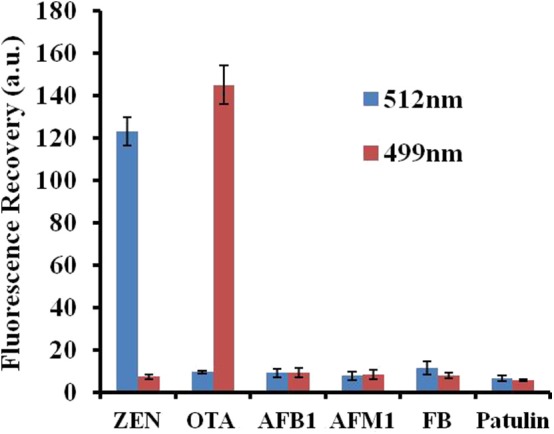
Selectivity assessment of the aptasensor. Error bars obtained from three parallel experiments.

The specificity of the fluorescence aptasensor was further checked using other possible interfering mycotoxins, such as aflatoxin B1 (AFB1), aflatoxin M1 (AFM1), fumonisin B1 (FB1), and patulin. ZEN, OTA, AFB1, AFM1, FB1, and patulin were added separately to the GO–APT1&2 complex at a concentration of 100 ng/ml each. The value of the fluorescence recovery is shown in Figure 6. The fluorescence recovery value of interfering mycotoxins was negligible, as shown in this bar chart. In comparison, the fluorescence recovery values of ZEN and OTA were apparent. The results strongly illustrated that APT1&2 possesses a higher specificity for ZEN and OTA, respectively, than other mycotoxins.

### Monitor of Dual Targets in Real Samples

Three different concentrations of ZEN and OTA were monitored by a fluorescence aptasensor. Reliability was further evaluated by recovery experiments in real samples using the standard spiking experiment. The concentrations of ZEN and OTA were determined using the method described above and calculated by using the linear equation. The result is shown in Table 2. The recoveries ranged from 89 to 99.89% for ZEN and from 91.75 to 103.56% for OTA. This method can be used to simultaneously check ZEN and OTA in real food samples.

**Table 2:** Real measurements of ZEN and OTA at different concentrations in wine samples.

**Wine sample**	**Added (ng/ml)**	**Founded (ng/ml)**	**Recovery (%)**
ZEN	1	0.89 ± 0.003	89
	4	3.85 ± 0.010	96.25
	16	15.95 ± 0.027	99.89
OTA	1	0.92 ± 0.007	92
	4	3.67 ± 0.015	91.75
	16	16.57 ± 0.039	103.56

### Information Steganography of the Fluorescence Aptasensor Based on GO

In this era of increasing information, the safety of messages has a crucial role. Information steganography is the method of hiding information (text, audio, video, and image data) within other information (text, audio, video, and image data). Moreover, information steganography is analogous to the natural camouflage of animals. There is no doubt that information steganography is a brilliant approach for the process of information transfer. It is very difficult to find hidden information within abundant information and to further remove the cover. Therefore, the information receiver must have the right key to decode hidden information. To further explore novel information steganography technology, DNA molecules and traditional steganography technology were combined in our work.

**Scheme 2:**
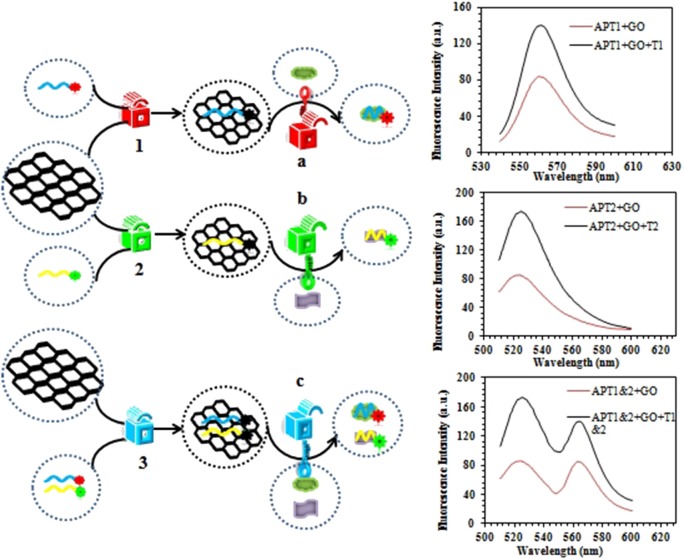
Schematic description of the graphene-based steganographic aptasensor for information encoding and steganography, which consists of GO as a cover, an aptamer as an information carrier, and ZEN/OTA as the special key.

The steganographic analysis relied mostly on the adsorption and release ability of GO for the ssDNA aptamer. The basic components of the steganography system (Scheme 2) included three parts: APT, GO, and dual targets (ZEN and OTA). The aptamer not only was an excellent information carrier (A, T, G, and C) but also bore a high specific recognition ability. In addition, GO could be recognized as a cover that could hide the information of an aptamer. Moreover, dual targets (ZEN and OTA) could be used as special keys that could decode the hidden information. In Scheme 1, the fluorescence quenching and ssDNA adsorption capability of GO were previously confirmed. This characteristic was applied to hide the information of the aptamer. Meanwhile, the quenched fluorescence signal could be used as an indicator signal that the aptamer information was successfully hidden by GO. In this procedure, GO played a role as a two-dimensional monoatomic layer platform and information cover. As we all know, a lock can only be opened by the correct key. In other words, the state of encrypted information would not change if there is no key or the correct key. Then, APT1 could specifically recognize ZEN, and APT2 could specifically recognize OTA. Therefore, the dual targets could be defined as the key to release the aptamer from GO and obtain the hidden information. The hidden information of APT1 could only be recovered by ZEN. Similarly, the hidden information of APT2 could only be recovered by OTA. The hidden double information of APT1 and APT2 could be recovered by ZEN and OTA. Thus, the key possessed high specificity for revealing the concealed information due to the high specificity of the aptamer to the target.

On a steganography foundation, the base sequences of the aptamer were further encrypted using complicated cipher texts. There are four possibilities (A, T, G, and C) for each DNA base position: the more bases of DNA there are, the higher the information density. We can use a few adjacent bases to represent letters or numbers and to further express the true information behind complicated cipher texts. Therefore, we can utilize information steganography and encryption technology to develop a dual-insurance method for information security.

## Conclusion

We designed a graphene-based steganographic aptasensor for multifunctional applications, simultaneous fluorescence detection of toxins (ZEN and OTA) of biofilm, and information computing, encryption, and concealment. Our constructed easy-to-use and efficient sensing platform for the simultaneous detection of ZEN and OTA may have wide application value in food and feed security monitoring. According to the change in materials and fluorescence, this graphene-based steganographic aptasensor can be utilized as a DNA-based encryption and concealment system for strengthening information security. This information steganographic system used an aptamer as an information carrier, GO as a cover, and targets as special keys. In summary, not only does our work offer a convenient simultaneous detection prototype for varied mycotoxins in foodstuff and feed safety monitoring, but our proposed model of a graphene-based steganographic aptasensor is also helpful for developing a molecular computer and information encryption and concealment technology.

## Data Availability Statement

The raw data supporting the conclusions of this article will be made available by the authors, without undue reservation, to any qualified researcher.

## Author Contributions

WW and QY were responsible for the conceptualization of the study, funding acquisition and the original draft. QW was responsible for the method, data acquisition, curation and analysis.

### Conflict of Interest

The authors declare that the research was conducted in the absence of any commercial or financial relationships that could be construed as a potential conflict of interest.
