# Supplementary material for: Graphene-Based Steganographic Aptasensor for Information Computing and Monitoring Toxins of Biofilm in Food
Source: Front Microbiol. 2020 Feb 4;10:3139. doi: 10.3389/fmicb.2019.03139 (PMC7010922; doi:10.3389/fmicb.2019.03139)
Supplement: Supplementary file 1 [file Table_1.pdf]

Supplementary Material

Table S1. Reported LOD for ZEN and OTA detection.

Target	Methods	LOD	Linear range	Ref.
ZEN	Molecularly imprinted polymers	2.09 ng /kg	6.25–250 ng /kg	Huang et al.
	Aptasensor	1 fg/mL	10 fg/mL-10 ng/mL	Luo et al.
	Lateral Flow biosensor	20 ng/mL	5–200 ng/mL	Wu et al.
	Aptasensor	0.5 ng/mL	0.5 to 64 ng/mL	Yugender et al.
	High performance liquid chromatography	5 μg/kg	None	Ren et al.
OTA	ELISA assay	40 pg/mL	5.0–640 pg/mL	Pei et al.
	Aptasensor	0.098 nM	None	Taghdisi et al.
	SPR sensor	18 pg/mL	None	Aleksandra et al.
	Immunosensor	14.86 pg/mL	0.015-0.4ng/mL	Li et al.

Reference:

Aleksandra Karczmarczyk, Ciril Reiner-Rozman, Simone Hageneder, Monika Dubiak-Szepietowska, & Karl-Heinz Feller. (2016). Fast and sensitive detection of ochratoxin a in red wine by nanoparticle-enhanced spr:. Analytica Chimica Acta, 937, 143-150.

Huang., Z., He, J., Li, Y., Wu, C., You, L., Wei, H., Li, K., & Zhang, S. (2019). Preparation of dummy molecularly imprinted polymers for extraction of Zearalenone in grain samples. J Chromatogr A, 1602, 11-18. doi:10.1016/j.chroma.2019.05.022

Li, M. , Liu, R. , Zhu, L. , & Chen, Z. . (2017). Development of ochratoxin a in cereal by chemiluminescence enzyme immunoassay. IOP Conference Series Earth and Environmental Science, 94(1), 012018.

Luo, L. J., Ma, S., Li, L. B., Liu, X. H., Zhang, J. Y., Li, X., Liu, D., & You, T. Y. (2019). Monitoring zearalenone in corn flour utilizing novel self-enhanced electrochemiluminescence aptasensor based on NGQDs-NH2-Ru@SiO2 luminophore. Food Chemistry, 292, 98-105. doi:10.1016/j.foodchem.2019.04.050

Pei, K., Xiong, Y., Xu, B., Wu, K., Li, X., Jiang, H., & Xiong, Y. (2018). Colorimetric ELISA for ochratoxin A detection based on the urease-induced metallization of gold nanoflowers. Sensors and Actuators B: Chemical, 262, 102-109. doi:https://doi.org/10.1016/j.snb.2018.01.193

Ren, DD., , Shao-Hui, L. I. , Yun-Feng, X. , & Yong-Tan, Y. . (2015). Improvement research on determination of zearalenonein grain by high performance liquid chromatography. Journal of Food Safety & Quality.

Taghdisi, S. M. , Danesh, N. M. , Beheshti, H. R. , Ramezani, M. , & Abnous, K. . (2016). A novel fluorescent aptasensor based on gold and silica nanoparticles for ultrasensitive detection of ochratoxin a. Nanoscale, 8(6), 3439-3446.

Wu, S., Liu, L., Duan, N., Li, Q., Zhou, Y., & Wang, Z. (2018). Aptamer-Based Lateral Flow Test Strip for Rapid Detection of Zearalenone in Corn Samples. Journal of Agricultural and Food Chemistry, 66(8), 1949-1954. doi:10.1021/acs.jafc.7b05326

Yugender Goud, K., Hayat, A., Satyanarayana, M., Sunil Kumar, V., Catanante, G., Vengatajalabathy Gobi, K., & Marty, J. L. (2017). Aptamer-based zearalenone assay based on the use of a fluorescein label and a functional graphene oxide as a quencher. Microchimica Acta, 184(11), 4401-4408. doi:10.1007/s00604-017-2487-6
